# Meta-analytic effect of *Saccharomyces cerevisiae* on dry matter intake, milk yield and components of lactating goats

**DOI:** 10.3389/fvets.2022.1014977

**Published:** 2022-11-18

**Authors:** Ifeanyi Princewill Ogbuewu, Christian Anayo Mbajiorgu

**Affiliations:** ^1^Department of Animal Science and Technology, Federal University of Technology, Owerri, Imo State, Nigeria; ^2^Department of Agriculture and Animal Health, University of South Africa, Florida, South Africa

**Keywords:** lactating goats, yeast products, supplementation, milk production traits, meta-analysis

## Abstract

The results of investigations on the impact of *Saccharomyces cerevisiae* (SC) on performance characteristics of lactating goats are inconsistent. Thus, this study aimed to summarize available evidence on the effect of SC supplementation on dry matter intake (DMI), milk yield and composition in lactating goats using meta-analysis. A systematic search performed on Scopus, Google Scholar and PubMed databases yielded 1,368 studies of which 18 were used for the meta-analysis. Subgroup and meta-regression analyses were performed to explore the sources of heterogeneity in response to dietary SC supplementation. A random-effects model showed that SC had a moderate effect on milk yield [standardized mean differences (SMD) = 0.51; 95% CI: 0.20 to 0.82, *p* = 0.001] and milk fat (SMD = 0.30; 95% CI: 0.05 to 0.55, *p* = 0.02) in lactating goats when compared to the controls. Subgroup analysis by SC type indicated that live SC had a large to moderate effect on milk yield (SMD = 1.46; 95% CI: 0.96 to 1.96, *p* < 0.001) and milk fat (SMD = 0.51; 95% CI: 0.19 to 0.84, *p* = 0.002), whereas dead SC had a large negative effect on DMI (SMD = −0.82; 95% CI: −1.28 to −0.7, *p* < 0.001) and a moderate reduction effect on milk yield (SMD = −0.55; 95% CI: −0.99 to −1.96, *p* = 0.015). We found significant heterogeneity across studies that evaluated the effect of SC treatment on DMI and milk yield in lactating goats and meta-regression analysis explained most of the sources of heterogeneity. In conclusion, pooled results showed that dietary SC supplementation increased milk yield and fat in lactating goats. In addition, subgroup analysis revealed that both live and fermented SC increased milk yield and fat in lactating goats, while dead SC reduced DMI and milk yield.

## Introduction

The demand for animal protein is on the increase in developing countries and this trend is expected to continue in the coming years (1, 2). Small ruminants, especially goats, have been recognized for their socio-economic and nutritional role to mankind for more than 7,000 years (3). The global goat population was over 1 billion in 2013 with a progressive increase of over 34% from 2,000 (4). Goats are essential for food security and play a significant role in the rural economy and livelihood sustenance (5). They have short generation interval and high fertility rate, making them economically important, especially for the rural farmers who drive goat production (6). Goats convert high fibrous feedstuffs that are undesirable for non-ruminants into high-quality milk and meat. Goat milk is more popular than cow milk, because of its superior nutritional properties, digestibility and sensory qualities (7). Goat milk is more medicinal than cow milk and contains fewer allergens (8).

There is a decrease in rumen activity during late gestation due to the pressure of the gravid uterus, resulting in lower DMI and thus loss of productivity (9). This calls for an increase in the nutrient requirements of goats during the pre-partum and post-partum periods using feed additives such as *Saccharomyces cerevisiae* (SC). Different types of SC products are commercially available, including live, dead and fermented SC products. Dead SC also called inactive contained only dead SC cells and it is grown primarily to express a certain nutrient, after which it is inactivated and harvested for that nutrient. Live SC works by scavenging oxygen in the rumen, stimulating the growth of fiber-degrading bacteria (10). It also produces metabolites that can be used as nutrients by rumen microbes (11). Although fermented SC products do not scavenge oxygen in the rumen, they do produce metabolites that boost rumen microbial activity, alter fermentation patterns, increase nutrient flow to the small intestine and improve digestion processes (11).

It is also reported that SC influences the rumen microbial population, resulting in changes in the level of volatile fatty acids (VFAs), which leads to an increase in DMI, milk yield and composition of lactating ruminants (12). DMI and milk production traits were found to be higher in some finding trials (13–16) and lower in others (17–19). The use of meta-analytic methods to aggregate published studies with variable results has gained attention in the field of animal agriculture in recent years (20, 21). To the best of our knowledge, no meta-analysis of the effect of SC on DMI and milk production traits in lactating goats has been published. Therefore, the purpose of this study was to use meta-analytic methods to evaluate available evidence on the effect of dietary SC supplementation on DMI, milk yield and composition in lactating goats.

## Materials and methods

### Literature search strategy

Scopus, Google Scholar and PubMed databases were searched for published studies that evaluated the impact of SC supplementation on DMI and milk production traits in lactating goats. The search terms included “dry matter intake,” “milk production,” “milk composition,” “milk yield” or “dietary *Saccharomyces cerevisiae*,” combined with “lactating goats”. The reference list of identified papers was searched for related studies. The article selection process adhered to the Preferred Reporting Items for Systematic Review and Meta-analysis (PRISMA) guidelines (22).

### Eligibility criteria

**Figure 1:**
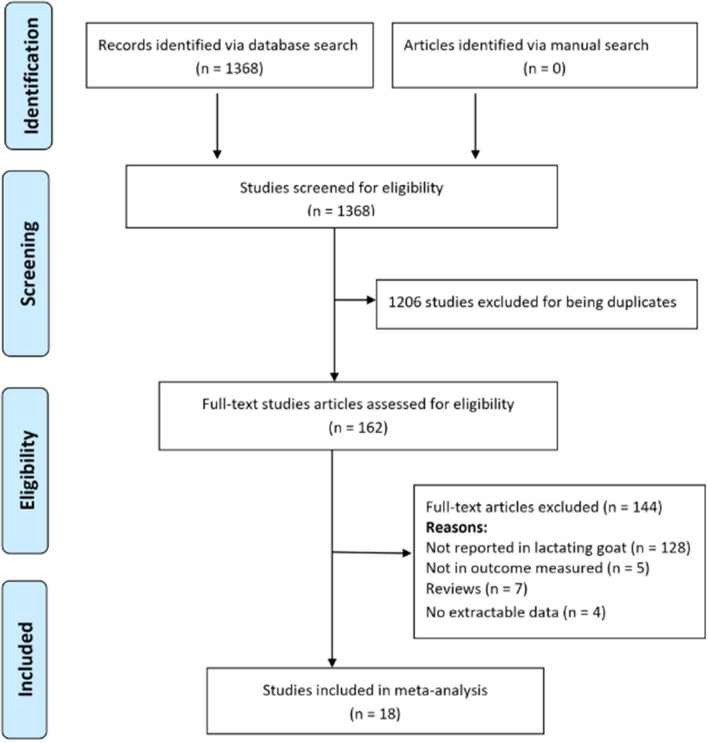
Flow chart of article selection process used for the meta-analysis.

Study selection was based on PICO criteria as shown in Table 1. Studies were included in the meta-analysis if, (i) the study assessed the effect of SC on DMI, milk yield, ash, proteins, lactose, total solids or fat in lactating goats, (ii) the experimental diets were free of antibiotics and other milk enhancing agents, (iii) the study has a control group fed diet without SC supplementation, and (iv) the study reported the mean, number of goats in each treatment group and a measure of variability such as standard error (SE), standard deviation (SD). The systematic search yielded 1368 publications. Based on title and abstract screening, 1,206 articles were excluded because they appeared in more than one database. One hundred and twenty-eight of the remaining 162 trials were excluded for not being in lactating goats. Out of the 34 studies remaining, seven were excluded for being a review and five for not being reported in our measured outcomes of interest. Four studies without sufficient data to calculate effect sizes were also excluded. Eighteen articles published in English met the selection criteria and were included in the meta-analysis (Figure 1). The included articles were independently assessed for eligibility and the debate as to include a study or not was resolved by consensus.

**Table 1:** PICO criteria.

	**Search strategy**	**Exclusion criteria**
Participant	Lactating goats	Non-lactating goats
Intervention	SC	Irrelevant treatment
Comparison	Control group (without SC supplementation)	
Outcomes	DMI, milk yield, protein, fat, lactose, total solids and ash	

SC, Saccharomyces cerevisiae; DMI, dry matter intake; PICO, population, intervention, comparison and outcomes.

### Data extraction

The following data were extracted from each of the 18 studies that met the inclusion criteria: the surname of the first author and year of publication. Data on the mean of measured outcomes (DMI, milk yield, protein, fat, ash, lactose or total solids) and the corresponding measures of variability, and the number of goats in the control and treatment groups were also extracted. Information on the following study characteristics was also extracted for meta-regression and subgroup analyses: location of study (South Africa, Saudi Arabia, Pakistan, Italy, Brazil, Egypt, Spain, China, India, France and Cyprus), type of SC (live, dead and fermented), breed (Beetal, Cilentana, Saanen, Zaraibi, Ardi, Nubian, Damascus, Murciano-Granadina, Alpine and Boar × local cross), treatment dose (0–22.9 g), diet type (mixed: concentrates + forages), treatment duration (<90 and ≥90 d), SC delivery methods (mixed or top dressed), method of SC feeding (individual or group), milking frequency (1 × daily milking, 2 × daily milking or 1 × weekly milking), initiation time of SC treatment (before or after kidding) and stage of lactation (early lactation: kidding to 90 days in milking, DIM; mid lactation: 91–180 DIM and late lactation: 181–270 DIM). SD was estimated from SE where it was not reported using the method described by Higgins and Deeks (23). Graphical data were extracted using WebplotDigitizer Version 4.5 designed by Rohatgi (24). In studies with multiple treatment comparisons, each treatment group was compared to a single control group using the methods described by Borenstein et al. (25). Table 2 summarized the characteristics of the 18 articles included in the study.

**Table 2:** Characteristics of studies included in the meta-analysis.

**s/no**	**Authors**	**Covariates**									
		**Location**	**SC type**	**Breed**	**TD**	**DT^1^**	**TDS**	**Delivery^2^**	**Feeding^3^**	**MF^4^**	**ITST^5^**	**SOL^6^**
1	Aaliya et al. (12)	India	Live	+++	0–4	Mixed	180	Mixed	1	1 ×	After	Late
2	Abd El-Ghani (13)	Egypt	Fermented	Zaraibi	0–6	Mixed	120	Mixed	1	1 × ^*^	After	Mid
3	Zicarelli et al. (14)	Italy	Fermented	Cilentana	0, 20	Mixed	120	TD	1	2 ×	After	Early
4	Abbas et al. (15)	RSA	Live	Beetal	0–10	Mixed	60	Mixed	1	2 ×	Before	Early
5	Khan et al. (16)	Pakistan	Live	Beetal	0–3	Mixed	45	Mixed	1	2 ×	After	Early
6	Lima et al. (17)	Brazil	Dead	Saanen	0–22.9	Mixed	90	Mixed	1	2 ×	After	Early
7	Gomes et al. (18)	Brazil	Dead	Saanen	0–2	Mixed	60	Mixed	1	2 ×	After	Early
8	Aazami et al. (19)	Italy	Fermented	Saanen	0–5	Mixed	22	Mixed	1	2 ×	After	Early
9	Mahrous et al. (26)	Egypt	Live	Zaraibi	0–3	Mixed	120	Mixed	1	2 ×	After	Mid
10	Salama et al. (27)	Spain	Fermented	+	0–1.5	Mixed	112	Mixed	1	1 ×	After	Mid
11	Stella et al. (28)	Italy	Live	Saanen	0–0.2	Mixed	105	Mixed	1	1 × ^*^	After	Early
12	Alshanbari et al. (29)	SA	Fermented	Ardi	0–4.5	Mixed	90	Mixed	1	1 ×	After	Early
13	Ma et al. (30)	China	Live	Saanen	0–5	Mixed	56	Mixed	1	2 ×	After	Early
14	Sahoo et al. (31)	India	Live	Beetal	0–2	nr	30	Mixed	1	2 ×	After	Early
15	Kholif et al. (32)	Egypt	Live	Nubian	0, 4	Mixed	22	Mixed	1	2 ×	After	Early
16	Ahmed et al. (33)	Egypt	Fermented	Zaraibi	0–2	Mixed	180	Mixed	1	1 ×	Before	Mid
17	Giger-Reverdin et al. (34)	France	Live	++	0–2	Mixed	42	Mixed	2	1 ×	After	Early
18	Hadjipanayiotou et al. (35)	Cyprus	Fermented	Damascus	0–5	Mixed	20	Mixed	2	2 ×	After	Early

TD, treatment dose; TDS, treatment duration of SC; DT, diet type; +, murciano-granadina; ++, alpine/saanen; +++, boar × local cross; ^*^1 × once weekly milking; nr, not reported; MF, milking frequency; SOL, stage of lactation; ITST, timing of SC treatment relative to calving; TD, top dressed; RSA, Republic of South Africa; SA, Saudi Arabia; 1, Individual feeding; 2, Group feeding.

1 Mixed concentrates with forages.

2 Mixed, treatment mixed in some portion of the feed; top-dressed, fed on top of the feed.

3 Individual indicates the lactating does were offered treatment on individual, while Group indicates that lactating does fed SC at the group level.

4 Number of times the study goats were milked in 24 h or in a week.

5 Timing of SC treatment relative to kidding.

6 Early lactation, kidding to 90 DIM (day in milking); mid lactation, 91–180 DIM and late lactation, 181–270 DIM.

### Statistical analysis

All analyses were performed in Open Meta-analyst for Ecology and Evolution (OpenMEE) software developed by Wallace et al. (36). A random effect model was used and results were analyzed as SMD at 95% CIs. SMD was classified as follows: low or small effect (0.2 |SMD| <0.5); moderate or medium effect: (0.5 |SMD| <0.8); large effect: (|SMD| ≥ 0.8) (37). Forest plots displayed the effect of SC treatment on DMI, milk yield and composition in lactating goats. SMD was considered significant when the lower and upper 95% CI did not include zero (38). Statistical heterogeneity (*p* < 0.05) among studies was assessed using DerSimonian and Laird test (Q-statistic) and quantified using the Inconsistency index (*I*^2^-statistic) (39). To investigate the sources of heterogeneity in response to SC treatment, meta-regression analyses were conducted on several key study characteristics. In the present study, we performed a meta-regression test in all measured outcomes since according to Baker et al. (40) a non-significant test for heterogeneity does not guarantee homogeneity among trials included in a meta-analysis. However, because of low statistical power, we did not perform meta-regression analyses in outcomes with less than 10 studies (41). Results of meta-regression analysis were considered significant at a 5% probability level. Subgroup analyses by type of SC, treatment dose, milking frequency, stage of lactation and treatment duration were performed to explore their influence on measured outcomes. Subgroups with fewer than 3 comparisons were excluded from the meta-analysis due to low statistical power (38). Sensitivity analysis was conducted to ascertain potential sources of heterogeneity as well as to assess the influence of excluding one study each time the analysis was performed (42). Publication bias was assessed graphically through funnel plot asymmetry. In the presence of publication bias, the number of trials required to reverse the reported findings (fail-safe number) was calculated based on Rosenberg method (43). Fail-safe number (Nfs) indicates the number of non-significant, unpublished (or missing) articles that will be required to reduce the overall statistically significant result to non-significant result. According to Jennions et al. (44), the results of a meta-analysis can be deemed robust despite the possibility of publication bias if Nfs is greater than 5^*^n + 10, where, *n* = number of trials included in the analysis.

## Results

### Study characteristics

The characteristics of 18 published trials used for the investigation are presented in Table 2. The flow chart of the study selection process is shown in Figure 1. A total of 11 and 17 trials assessed the effect of SC on DMI and milk yield in lactating goats respectively. Furthermore, 16, 15, 13, 7 and 11 studies examined the relationship between SC supplementation and milk composition (protein, fat, lactose, ash and total solids, respectively). The included studies were conducted in eleven countries, majority in Egypt (*n* = 4), Italy (*n* = 3), India (*n* = 2) or Spain (*n* = 2). Most trials were conducted on live yeast (*n* = 9), followed by fermented SC (*n* = 7) and dead SC (*n* = 2). The included studies were published between 1996 and 2020, covering 23 years. The most commonly studied breeds were Saanen (*n* = 6), Beetal (*n* = 3) and Zaraibi (*n* = 3). Treatment dose and treatment duration ranged from 0 to 22.9 g/day/animal and 22 to 189 days, respectively. The majority of the studies included in the meta-analysis fed SC to goats after kidding.

### Results of pooled analysis

**Figure 2:**
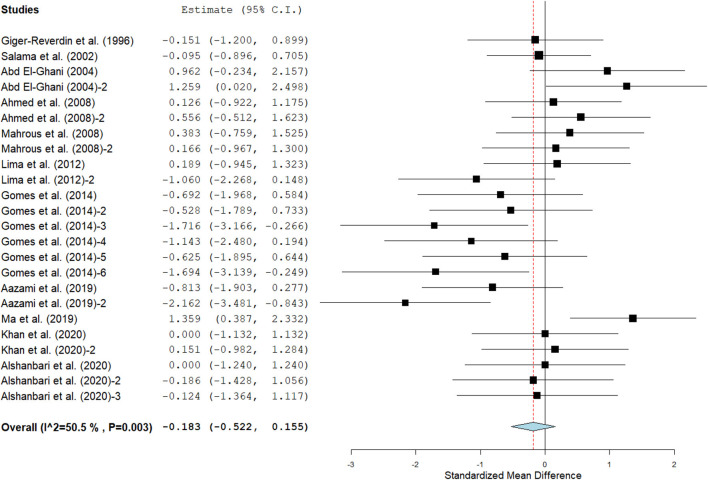
Forest plot of SMD for DMI in lactating goats. The error bars (black square boxes) connote the SMD of each trial, while the upper and lower 95% confidence intervals (CIs) for the effect size are the line that joined the individual square box. The thick vertical line is the line of no effect (SMD = 0), which suggests no effect of SC supplementation on milk yield. The sky-blue diamond at the base of the plot indicates the pooled SMD with its width representing the 95% confidence intervals for the effect size. The points to the **right** of the line of no effect suggest an increase in DMI. The points to the **left** of the line of no effect connote an increase in DMI. *I*^2^ is the inconsistency index.

**Figure 3:**
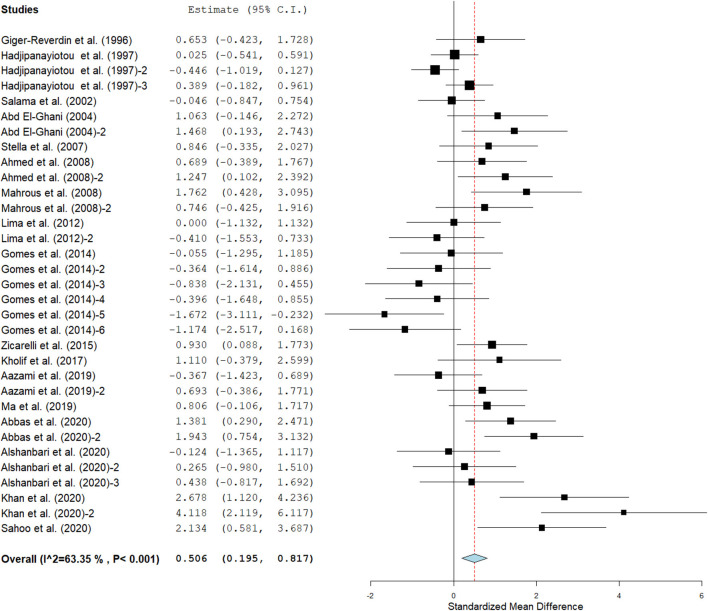
Forest plot of the impact of SC on milk yield in lactating goats. The error bars connote the SMD of each trial, while the upper and lower 95% CIs for the effect size are the line that joined the individual square box. The thick vertical line is the line of no effect (SMD = 0), which suggests no effect of SC supplementation on milk yield. The sky-blue diamond at the base of the plot indicates the pooled SMD with its width representing the 95% confidence intervals for the effect size. The points to the **right** of the line of no effect suggest an increase in milk yield. The points to the **left** of the line of no effect connote an increase in milk yield. *I*^2^ is the inconsistency index.

**Figure 4:**
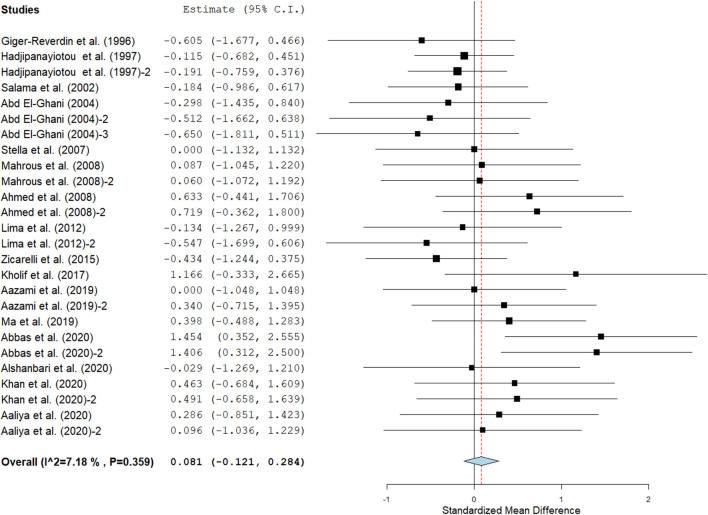
Effect of SC on milk proteins in lactating does. The error bars connote the SMD of each trial, while the upper and lower 95% CIs for the effect size are the line that joined the individual square box. The thick vertical line is the line of no effect (SMD = 0), which suggests no effect of SC treatment on milk proteins. The sky-blue diamond at the base of the plot indicates the pooled SMD with its width representing the 95% confidence intervals for the effect size. The points to the **right** of the line of no effect suggest an increase in milk proteins. The points to the **left** of the line of no effect connote an increase in milk proteins. *I*^2^ is the inconsistency index.

**Figure 5:**
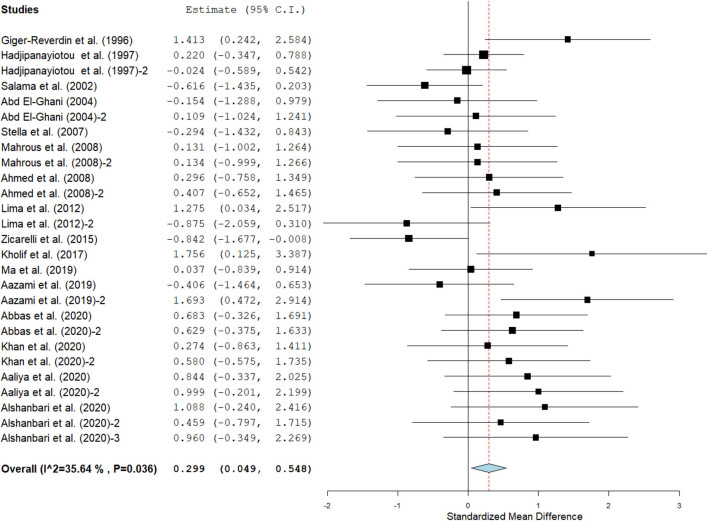
Influence of SC on milk fat yield in lactating goats. The error bars connote the SMD of each trial, while the upper and lower 95% CIs for the effect size are the line that joined the individual square box. The thick vertical line is the line of no effect (SMD = 0), which suggests no effect of dietary SC intervention on milk fat yield. The sky-blue diamond at the base of the plot indicates the pooled SMD with its width representing the 95% confidence intervals for the effect size. The points to the **right** of the line of no effect suggest an increase in milk fat yield. The points to the **left** of the line of no effect connote an increase in milk fat yield. *I*^2^ is the Inconsistency index.

**Figure 6:**
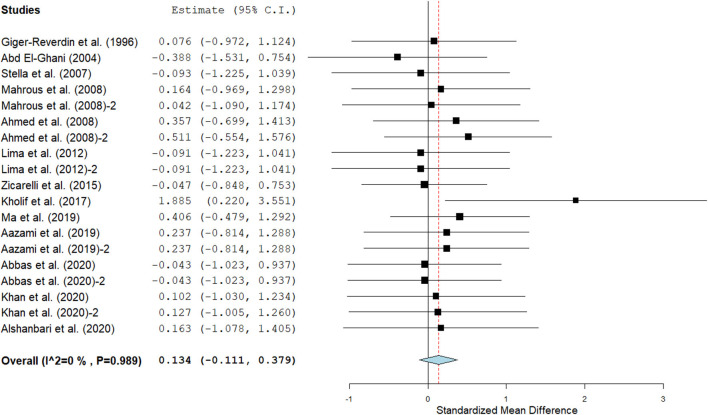
Forest plot of the effect of SC on milk lactose in lactating goats. The error bars connote the SMD of each trial, while the upper and lower 95% CIs for the effect size are the line that joined the individual square box. The thick vertical line is the line of no effect (SMD = 0), which suggests no effect of SC supplementation on milk lactose. The sky-blue diamond at the base of the plot indicates the pooled SMD with its width representing the 95% confidence intervals for the effect size. The points to the **right** of the line of no effect suggest an increase in milk lactose. The points to the **left** of the line of no effect connote an increase in milk lactose. *I*^2^ is the inconsistency index.

**Figure 7:**
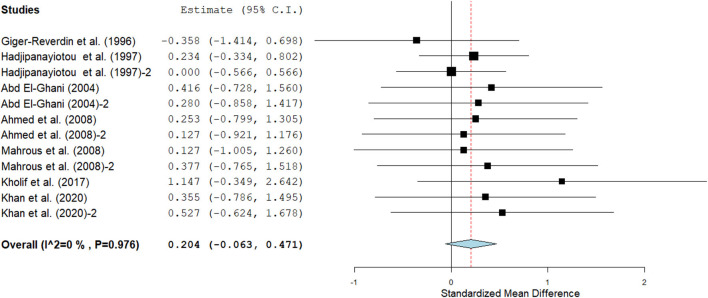
The effect of SC on milk ash content in lactating goats. The error bars connote the SMD of each trial, while the upper and lower 95% CIs for the effect size are the line that joined the individual square box. The thick vertical line is the line of no effect (SMD = 0), which suggests no effect of SC supplementation on milk ash. The sky-blue diamond at the base of the plot indicates the pooled SMD with its width representing the 95% confidence intervals for the effect size. The points to the **right** of the line of no effect suggest an increase in milk ash, while the points to the **left** of the line of no effect connote an increase in milk ash. *I*^2^ is the inconsistency index.

**Figure 8:**
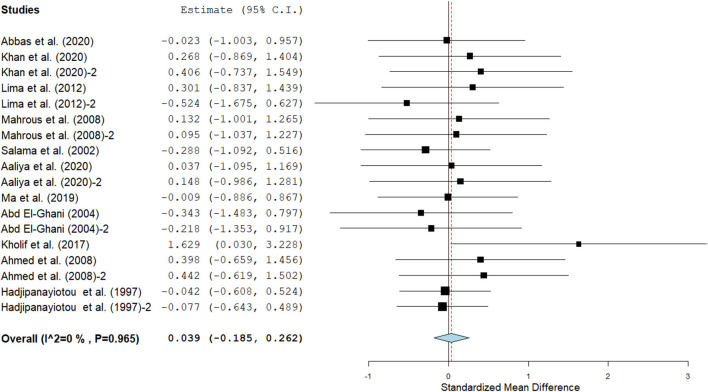
The forest plot of the effect of SC on milk total solids in lactating goats. The error bars connote the SMD of each trial, while the upper and lower 95% CIs for the effect size are the line that joined the individual square box. The thick vertical line is the line of no effect (SMD = 0), which suggests no effect of SC supplementation on milk total solids. The sky-blue diamond at the base of the plot indicates the pooled SMD with its width representing the 95% confidence intervals for the effect size. The points to the **right** of the line of no effect suggest an increase in milk total solids. The points to the **left** of the line of no effect connote an increase in milk total solids. *I*^2^ is the inconsistency index.

Results show that SC treatment had a small effect on DMI (SMD = −0.18, 95% CI: −0.52 to 0.16, *p* = 0.289; Figure 2) and a moderate effect on milk yield (SMD = 0.51, 95% CI: 0.20 to 0.82, *p* = 0.001; Figure 3) in lactating goats when compared to the controls. Dietary SC supplementation had a small treatment effect on milk proteins (SMD = 0.08, 95% CI: −0.12 to 0.28, *p* = 0.431; Figure 4), fat (SMD = 0.30, 95% CI: 0.05 to 0.55, *p* = 0.019; Figure 5), lactose (SMD = 0.13, 95% CI: −0.11 to 0.38, *p* = 0.283; Figure 6), ash (SMD = 0.20, 95% CI: −0.06 to 0.47, *p* = 0.301; Figure 7) and total solids (SMD = 0.04, 95% CI: −0.19 to 0.26, *p* = 0.734; Figure 8).

### Results of subgroup analyses

#### DMI and milk yield

Results of subgroup analysis of the influence of SC on DMI and milk yield are presented in Table 3. Subgroup analysis of DMI by SC type showed that live and fermented SC had a small treatment effect on DMI in lactating goats, while dead SC had a large negative effect on DMI (SMD = −0.82, 95% CI: −1.28 to −0.37, *p* < 0.001) in lactating goats. Subgroup analysis of DMI for treatment duration indicated that lactating goats fed SC for <90 days had moderately lower DMI compared to controls. In contrast, treatment dose, milking frequency and stage of lactation had a small impact on DMI in lactating goats.

**Table 3:** Effect of covariates on DMI and milk yield of lactating goats fed SC supplemented diets.

**Outcomes**	**Covariates**	***n***	**SMD**	**95% CI**		**SE**	***p*-val**	**Heterogeneity**	
				**Lower**	**Upper**			***I^2^***	**p-val**
**DMI**	**SC type**
	Live	6	0.36	−0.11	0.84	0.24	0.136	11	0.347
	Dead	8	−0.82	−1.28	−0.37	0.23	<0.001	0	0.469
	Fermented	10	−0.03	−0.57	0.50	0.27	0.906	55	0.017
	**Treatment dose**
	<5 g/animal/day	19	−0.20	−0.49	0.08	0.15	0.162	13	0.295
	≥5 g/animal/day	5	−0.05	−1.35	1.24	0.66	0.937	84	<0.001
	**MF**
	1 × daily milking	7	0.02	−0.38	0.42	0.21	0.927	0	0.969
	2 × daily milking	15	−0.48	−0.96	0.01	0.25	0.054	59	0.002
	**SOL**
	Early lactation	13	−0.26	−0.72	0.19	0.23	0.256	49	0.024
	Mid lactation	9	0.11	−0.43	0.64	0.27	0.694	50	0.044
	**Treatment duration (d)**
	<90	12	−0.60	−1.17	−0.03	0.29	0.040	62	0.002
	≥90	12	0.17	−0.16	0.49	0.16	0.315	0	0.504
**Milk yield**	**SC type**
	Live	11	1.46	0.96	1.96	0.26	<0.001	42	0.069
	Dead	8	−0.55	−0.99	−0.11	0.23	0.015	0	0.651
	Fermented	14	0.34	0.03	0.65	0.16	0.030	37	0.079
	**Treatment dose**
	<5 g/animal/day	22	0.49	0.05	0.94	0.23	0.031	65	<0.001
	≥5 g/animal/day	11	0.53	0.10	0.96	0.22	0.017	63	0.002
	**MF**
	1 × daily milking	7	0.40	−0.01	0.81	0.21	0.054	0	0.607
	2 × daily milking	23	0.47	0.05	0.88	0.21	0.027	72	<0.001
	**SOL**
	Early lactation	22	0.60	0.24	0.97	0.19	0.001	63	<0.001
	Mid lactation	9	0.61	0.06	1.15	0.28	0.030	51	0.039
	**Treatment duration (d)**
	<90	19	0.45	−0.02	0.92	0.24	0.062	74	<0.001
	≥90	14	0.59	0.26	0.92	0.17	<0.001	16	0.274

n, comparisons; d, day; g, grams; SOL, stage of lactation; MF, milking frequency; DMI, dry matter intake; DIM, day in milking; SMD, standardized mean difference; CI, confidence interval; SE, standard error; I^2^, Inconsistency index; p-val, probability value.

Milk yield (Table 3) results showed a significant treatment effect when the analysis was stratified by SC types. Results revealed that live SC had a large positive effect in milk yield (SMD = 1.46, 95% CI: 0.96 to 1.96, *p* < 0.001), while fermented SC had a small effect on milk yield (SMD = 0.34, 95% CI: 0.03 to 0.65, *p* = 0.030; *I*^2^ = 37%, *p* = 0.079) in lactating goats when compared to the controls. In contrast, dead SC had a moderate negative effect on milk yield (SMD = −0.55, 95% CI: −0.99 to −0.11, *p* = 0.015; *I*^2^ = 0%). SC intervention had a significant effect on milk yield when the analysis was stratified by stage of lactation. Stage of lactation had a positive and medium effect on milk yield (early-lactation: SMD = 0.60, 95% CI: 0.24 to 0.97, *p* = 0.001) and mid-lactation: SMD = 0.61, 95% CI: 0.06 to 1.15, *p* = 0.030). In the analysis stratified by treatment dose, a statistically significant moderate positive effect was noted between treatment dose at ≥5 g/day/animal and milk yield (SMD = 0.53, 95% CI: 0.10 to 0.96, *p* = 0.017). However, treatment dose at <5 g/day/animal had a statistically small positive effect on milk yield (SMD = 0.49, 95% CI: 0.05 to 0.94, *p* = 0.031) in lactating goats. Subgroup analysis of milk yield by milking frequency showed that both 1 × daily milking and 2 × daily milking had a small positive influence on milk yield (1 × daily milking: SMD = 0.40, 95% CI: −0.01 to 0.81, *p* = 0.054) and 2 × daily milking: SMD = 0.47, 95% CI: 0.05 to 0.88, *p* = 0.027). In converse, lactating goats fed SC for ≥ 90 days had moderately higher milk yield (SMD = 0.59, 95% CI: 0.26 to 0.92, *p* < 0.001) than controls. The forest plots of DMI (Figure 2) and milk yield results (Figure 3) show evidence of significant heterogeneity (DMI: *I*^2^– statistic = 51%, *p* = 0.003 and milk yield: *I*^2^ = 63%, *p* < 0.001, respectively), which subgroup analyses could not completely resolve.

#### Milk composition

Results of subgroup analyses of milk proteins and fat are presented in Table 4, while milk lactose and total solids results are summarized in Table 5. Subgroup analysis of milk proteins stratified by SC types revealed that live SC (SMD = 0.42, 95% CI: 0.07 to 0.76, *p* = 0.018) and fermented SC (SMD = −0.01, 95% CI: −0.35 to 0.15, *p* = 0.444) had a small effect on milk proteins. The magnitude effect sizes for milk proteins were low when the analysis was stratified by treatment dose milking frequency, stage of lactation and treatment duration. Subgroup analysis of milk fat stratified by SC types shows that live SC had a moderate effect on milk fat (SMD = 0.51, 95% CI: 0.19 to 0.84, *p* = 0.002). Subgroup analysis of milk fat percentage showed a significant treatment effect when the analysis was stratified by milking frequency. 1 × daily milking had a moderate positive effect on milk fat in lactating does (SMD = 0.56, 95% CI: 0.10 to 1.01, *p* = 0.018) compared to the controls. The forest plot of milk fat results (Figure 5) found significant heterogeneity across studies (*I*^2^ – statistic = 36%, *p* = 0.036), which subgroup analysis could not completely resolve. Treatment dose, stage of lactation and treatment duration had a marginal effect on milk fat yield in lactating goats. Results of subgroup analysis by milk lactose and total solids revealed that treatment dose, milking frequency, stage of lactation and treatment duration had a small effect on milk lactose and total solids in lactating goats. We did not conduct subgroup analysis for milk ash because of insufficient data.

**Table 4:** Effect of covariates on milk protein and fat of lactating goats fed SC supplemented diets.

**Outcomes**	**Covariates**	***n***	**SMD**	**95% CI**		**SE**	***p*-val**	**Heterogeneity**	
				**Lower**	**Upper**			***I^2^***	***p*-val**
**Milk protein**	**SC type**
	Live	12	0.42	0.07	0.76	0.18	0.018	13	0.320
	Fermented	12	−0.10	−0.35	0.15	0.13	0.444	0	0.801
	**Treatment dose**
	<5 g/animal/day	15	0.14	−0.14	0.43	0.15	0.327	0	0.903
	≥5 g/animal/day	11	0.06	−0.32	0.44	0.19	0.750	47	0.043
	**MF**
	1 × daily milking	7	0.10	−0.30	0.50	0.20	0.625	0	0.609
	2 × daily milking	15	0.19	−0.10	0.48	0.15	0.198	27	0.156
	1 × weekly milking	4	−0.36	−0.93	0.21	0.29	0.218	0	0.872
	**SOL**
	Early lactation	16	0.14	−0.15	0.43	0.15	0.330	29	0.135
	Mid lactation	8	−0.02	−0.39	0.36	0.19	0.938	0	0.600
	**DOS (d)**
	<90	11	0.32	−0.05	0.70	0.19	0.092	42	0.069
	≥90	15	−0.08	−0.35	0.20	0.14	0.586	0	0.914
**Milk fat**	**SC type**
	Live	12	0.51	0.19	0.84	0.16	0.002	0	0.557
	Fermented	13	0.13	−0.21	0.48	0.18	0.446	41	0.061
	**Treatment dose**
	<5 g/animal/day	16	0.37	0.04	0.70	0.17	0.028	24	0.187
	≥5 g/animal/day	11	0.22	−0.17	0.61	0.20	0.270	50	0.029
	**MF**
	1 × daily milking	9	0.56	0.10	1.01	0.23	0.018	33	0.157
	2 × daily milking	15	0.25	−0.09	0.59	0.17	0.153	45	0.032
	**SOL**
	Early lactation	18	0.37	0.04	0.71	0.17	0.030	48	0.012
	Mid lactation	7	−0.02	−0.41	0.38	0.20	0.927	0	0.777
	**Treatment duration (d)**
	<90	11	0.46	0.10	0.82	0.19	0.013	37	0.104
	≥90	16	0.16	−0.18	0.51	0.18	0.352	34	0.089

d, day; g, grams; SOL, stage of lactation; MF, milking frequency; DIM, day in milking; SMD, standardized mean difference; CI, confidence interval; SE, standard error; I^2^, Inconsistency index; p-val, probability value.

**Table 5:** Effect of covariates on milk lactose and total solids of lactating goats fed SC supplemented diets.

**Outcomes**	**Covariates**		**SMD**	**95% CI**		**SE**	***p*-val**	**Heterogeneity**	
				**Lower**	**Upper**			***I^2^***	***p*-val**
**Milk lactose**	**SC type**
	Live	10	0.17	−0.17	0.51	0.18	0.332	0	0.833
	Fermented	7	0.14	−0.25	0.53	0.20	0.475	0	0.943
	**Treatment dose**
	<5 g/animal/day	12	0.20	−0.13	0.52	0.17	0.244	0	0.886
	≥5 g/animal/day	7	0.06	−0.31	0.423	0.19	0.757	0	0.986
	**MF**
	1 × daily milking	4	0.28	−0.26	0.83	0.28	0.310	0	0.944
	2 × daily milking	13	0.14	−0.15	0.43	0.15	0.342	0	0.941
	**SOL**
	Early lactation	14	0.13	−0.16	0.41	0.14	0.375	0	0.959
	Mid lactation	5	0.15	−0.34	0.65	0.25	0.540	0	0.830
	**DOS (d)**
	<90	7	0.22	−0.13	0.58	0.18	0.216	0	0.789
	≥90	10	0.05	−0.29	0.39	0.17	0.763	0	0.993
**Milk total solids**	**SC type**
	Live	9	0.20	−0.17	0.56	0.19	0.299	0	0.878
	Fermented	7	−0.05	−0.35	0.26	0.15	0.769	0	0.900
	**Treatment dose**
	<5 g/animal/day	11	0.17	−0.17	0.50	0.17	0.326	0	0.822
	≥5 g/animal/day	7	−0.07	−0.37	0.24	0.16	0.661	0	0.981
	**MF**
	1 × daily milking	5	0.09	−0.36	0.54	0.23	0.692	0	0.806
	2 × daily milking	11	0.06	−0.22	0.33	0.14	0.686	0	0.835
	**SOL**
	Early lactation	7	0.05	−0.24	0.34	0.15	0.741	0	0.677
	Mid lactation	7	0.01	−0.39	0.40	0.20	0.971	0	0.893
	**DOS (d)**
	<90	7	0.07	−0.24	0.38	0.16	0.651	0	0.600
	≥ 90	11	0.03	−0.32	0.323	0.17	0.986	0	0.970

d, day; g, grams; MF, milking frequency; SOL, stage of lactation; SMD, standardized mean difference; CI, confidence interval; SE, standard error; I^2^, Inconsistency index; p-val, probability value.

#### Analysis moderators and publication bias

To examine sources of heterogeneity, we conducted meta-regression analysis on the following study characteristics: location of study, SC types, breed, treatment dose, treatment duration, SC delivery methods, methods of SC feeding, milking frequency, initiation time of SC treatment and stage of lactation. We did not perform meta-regression analysis on diet type because 17 studies out of the 18 that met the inclusion criteria blended concentrate with forage and one study did not state the diet type used. Results of meta-regression variables influencing the effect size of SC on DMI, milk yield and proteins are presented in Table 6. SC type (*p* = 0.008), milking frequency (*p* = 0.008), treatment duration (*p* = 0.008) and study location (*p* < 0.001) were predictors of treatment effect on DMI. The studied covariate accounted for most of the sources of heterogeneity. SC types, stage of lactation, breed and study location were significant predictors of study effect on milk yield. There was also a positive significant relationship between milk proteins and prediction variables (SC type, breed and initiation time of SC treatment). Results (Table 7) showed that breed (*p* = 0.003) and SC delivery method (*p* = 0.024) were significant predictors of the impact of SC on milk fat. Prediction variables were not significant predictors of the treatment effect on milk lactose and total solids.

**Table 6:** Relationships between covariates and outcome measures (DMI, milk yield and proteins).

**Outcomes**	**Covariates**	**Q_M_**	***p*-value**	***R*^2^-index (%)**
DMI	SC type	9.64	0.008	49
	Treatment dose	0.33	0.568	0
	Milking frequency	7.46	0.023	32
	Stage of lactation	3.48	0.176	11
	Treatment duration	5.05	0.025	21
	Breed	9.83	0.080	19
	Feeding	0.98	0.965	0
	Location	34.5	1.38e-05	100
	ITST	0.92	0.338	0
Milk yield	SC type	35.8	1.68e-08	81
	Treatment dose	0.05	0.830	0
	Milking frequency	1.26	0.533	0
	Stage of lactation	8.42	0.015	25
	Treatment duration	0.29	0.59	0
	Breed	47.4	1.29e-07	86
	Feeding	1.07	0.301	1
	Location	67.7	1.22e-10	98
	Delivery	0.24	0.628	0
	ITST	3.80	0.051	20
Milk proteins	SC type	7.10	0.022	100
	Treatment dose	0.42	0.515	17
	Milking frequency	2.49	0.287	0
	Stage of lactation	0.36	0.835	0
	Treatment duration	2.34	0.126	0
	Breed	16.8	0.052	100
	Feeding	2.92	0.088	100
	Location	17.9	0.057	100
	ITST	14.1	<0.001	100

DMI, dry matter intake; ITST, initiation time of SC treatment relative to kidding; R^2^, amount of heterogeneity accounted for by covariate; Q_M_, coefficient of moderators; p-value, probability value.

**Table 7:** Relationships between covariates and outcome measures (milk fat, lactose and total solids).

**Outcomes**	**Covariates**	***Q*_M_**	***p*-value**	***R*^2^-index (%)**
Milk fat	SC type	2.59	0.273	18
	Treatment dose	0.42	0.518	0
	Milking frequency	2.35	0.308	0
	Stage of lactation	3.05	0.218	9
	Treatment duration	1.45	0.228	0
	Breed	24.7	0.003	100
	Feeding	0.03	0.863	0
	Location	13.1	0.22	45
	Delivery	5.11	0.024	47
	ITST	0.45	0.484	0
Milk lactose	SC type	0.35	0.841	0
	Treatment dose	0.30	0.586	0
	Milking frequency	1.12	0.572	0
	Stage of lactation	0.01	0.927	0
	Treatment duration	0.47	0.494	0
	Breed	4.63	0.592	0
	Feeding	0.01	0.91	0
	Location	1.44	0.984	0
	Delivery	0.22	0.64	0
	ITST	0.03	0.853	0
Milk total solids	SC type	1.11	0.573	0
	Treatment dose	1.04	0.308	0
	Milking frequency	0.67	0.715	0
	Stage of lactation	0.05	0.977	0
	Treatment duration	0.09	0.765	0
	Breed	5.09	0.532	0
	Feeding	0.34	0.563	0
	Location	2.18	0.949	0
	ITST	0.60	0.439	0

ITST, initiation time of SC treatment relative to kidding; R^2^, amount of heterogeneity accounted for by covariate; QM, coefficient of moderators; p-value, probability value.

Visual inspection of funnel plots showed little asymmetry for trials on the effect of SC on milk yield and fat in lactating goats (Supplementary Figures S1, S2). The Rosenberg Nfs for the database were 780 for milk yield and 650 for milk fat. These values were four times greater than the threshold of 175 (5 × *n* = 33 + 10) and 145 (5 × *n* = 27 + 10) needed to declare the mean effect size of outcomes (milk yield and fat, respectively) robust. Sensitivity analyses by dropping one study each time the analysis was conducted did not substantially alter the pooled results.

## Discussion

### Effect of SC on DMI and milk production characteristics

The present meta-analysis is the first to explore the effect of dietary SC supplementation on DMI and milk production traits in lactating goats. We found that SC treatment moderately increased milk yield and had no effect on DMI and milk components in lactation goats. The small effect of SC treatment on DMI in the present study agrees with the findings of Rossow et al. (45) who discovered that SC had no effect on DMI in cows. Although the mechanism by which SC improves milk yield and components in lactating goats is not well known. The moderate positive effect of SC supplementation on milk yield in lactating goats might be related to the ability to enhance the growth of cellulolytic bacteria in the rumen, leading to an increase in total ruminal VFAs and propionate (C3) levels while decreasing acetate (C2), butyrate (C4) and the C2 to C3 ratio (46). In a similar study on cows, Zhang et al. (47) found that dietary propionic acid had a positive effect on milk performance characteristics. The results of this meta-analysis are consistent with the findings of Khan et al. (16), who observed higher milk yield in lactating goats fed SC at 1.5 and 3.0 g/day/animal than that fed diet without SC supplementation.

### Stratification and analysis of moderators

#### Types of *Saccharomyces cerevisiae*

There is a correlation between nutrition and milk fat percentage in ruminants, with nutrition accounting for approximately 50% of the differences in milk fat content (8). This meta-analysis found that SC type is a significant predictor of the study effect, with lactating goats offered live SC having higher milk yield and fat percentage than the controls. These results are not in agreement with the findings of Khan et al. (16), who reported 34–59 and 3–6% increase in milk yield and fat, respectively in lactating Beetal goats fed live SC at 1.5–3.0 g/day/head when compared to those fed diet without SC supplementation. It has been shown that energy status of the dam affects milk yield and its constituents in farm animals (8). The exact mechanism underlying the observed increase in milk yield and fat content in lactating goats offered live SC in the current study is not clear. However, this could be attributed to the capability of SC to scavenge excess oxygen in the ruminal fluids, lower the redox potential and enhance the growth of cellulolytic bacteria, leading to an increase in milk yield and fat content. It is also well known that the addition of live SC to the diets of lactating goats optimizes ruminal VFAs proportions and lowers the C3 to C2 ratios, which might be a contributing factor to the improved milk yield in the present study (48). The moderate positive effect of live SC on milk fat yield as recorded in the present study could be related to the ability of SC to lower ruminal C2 level, a precursor in milk fat synthesis (11).

Our results show that SC types are significant predictors of intervention effect on DMI, milk yield and proteins and accounted for the majority of the between-study variance. The moderate to large reduction effect of dead SC on DMI and milk yield in lactating goats in the present study implies that dead SC may not support the growth of cellulolytic bacteria in the rumen. The exact mechanisms that lead to the negative effect of dead SC on DMI and milk yield in lactating goats are not clear. This could be due to the ability of dead SC to increase lactate accumulation in the rumen, raise the concentrations of dissolved oxygen in the ruminal fluid, and decrease the utilization of dietary starch. In this way, dead SC reduces the rate of VFAs production and, hence, lowers the stability of the rumen environment and intensity of fiber degradation, which may result in lower DMI and milk yield (49).

#### Treatment dose and milking frequency

The moderate effect of high doses of SC on milk yield implies that future research should be directed at determining the supplementation levels of SC that support optimum milk yield in lactating goats. In this study, DMI, milk yield and proteins for 2 × daily milking were higher than 1 × daily milking. Our results are consistent with the findings of Williams et al. (50), who reported that milk yield and DMI increase with milking frequency in lactating goats. This could be explained in terms of milking frequency to increase mammary epithelial cell (MEC) number, reduce MEC apoptosis, increase cell activity and concentration of putative feedback inhibitor of lactation (FIL) from the mammary glands. Although milking frequency stimulates mammary functions and milk synthesis, the likely interactions between SC supplementation and milking frequency in this study cannot be ruled out. The increase in the concentration of putative FIL synthesized by the mammary gland and intra-mammary pressure may cause the decrease in milk yield in 1 × daily milking in the present study. On the same hand, the loss of tight junction integrity after about 20 h of milk accumulation may play a role in milk yield losses in 1 × daily milking (50).

The moderately higher milk yield in goats fed live SC at a high level compared to controls supports the findings of Abd El-Ghani (13) and Masek et al. (51), who reported dose-related increases in milk yield in lactating small ruminants fed diets containing high levels of live SC. This might be attributed to the ability of SC to improve the growth of cellulolytic bacteria, which resulted in higher fiber digestion and enhanced production of total VFAs, thus allowing higher energy availability for milk yield. This finding is in agreement with the results of others who reported that SC increases fiber degradation and optimizes VFAs proportions in the rumen (10). There is no relationship between treatment dose and measured outcomes, implying that treatment dose is not a significant predictor of the effect of SC intervention on DMI, milk yield and components in lactating goats. The moderately higher milk fat content in 1 × daily milking is consistent with the findings of Løvendahl and Chagunda (52), who reported that 1 × daily milking reduces milk yield while increasing milk fat in cows. This study found that milking frequency accounted for 32% of SC intervention on DMI. The lack of a significant association between milking frequency and aspects of our outcome measures implies that milking frequency is not a good predictor of the study effect.

#### Stage of lactation and treatment duration

Stage of lactation is a limiting factor in this meta-analysis and explained 25% of the sources of variability across studies that assessed the effect of SC treatment on milk yield in lactating goats. Our results show that treatment duration had a moderate positive effect on milk yield in lactating goats. This observation may be related to the ability of SC to provide important nutrients or nutritional co-factors that stabilizes rumen pH, improve fermentation and encourage the growth of lactate-utilizing bacteria. In the rumen, fiber digestibility and utilization are enhanced when SC is added to the rations, resulting in an increase in milk yield (11). In a similar meta-analysis, Poppy et al. (53) reported an increase in milk yield in cows fed SC supplemented diets. The present study shows that long-term SC treatment had a medium effect on milk yield. This may be related to the ability of SC supplemented diets to continuously meet the nutritional requirements imposed for high levels of milk production in lactating goats.

#### Breed, study location, initiation time of sc treatment and sc delivery method

This study found that Damascus, Cilentana and Murciano-Granadina goats fed fermented SC had lower milk yield when compared to controls. One possible biochemical mechanism for the decrease in milk yield on these breeds is the poor ability of fermented SC to stimulate rumen microbial activities, resulting in a decrease in milk yield. However, Beetal goats fed live SC had a higher milk fat percentage than controls. The mechanism of action was that live SC increased ruminal acetate production, a which is a precursor of milk fat synthesis (54). These results confirmed the findings of Lopez-Villalobos et al. (55), who found a correlation between breed and milk production traits (milk yield and composition) in cows. Location is a limiting factor in this meta-analysis and explained 98–100% of the variability in DMI and milk yield in lactating goats. Saanen goats reared on live SC in China had higher DMI, while the same goat reared on live or fermented SC in Italy had lower DMI. In addition, Saanen goats fed dead SC in Brazil had higher milk yield, whereas Beetal goats fed live SC in Pakistan had lower milk yield. These differences could be attributed to seasonal variation found to affect DMI and milk composition in cows (56). Results showed that SC delivery method and initiation time of SC treatment had a medium to large effect on milk fat and proteins, explaining 45% and 100% of the between-study variance in milk fat and proteins, respectively.

#### Publication bias

Publication bias is a common problem in meta-analysis, as it may alter the pooled effect estimate of SC treatment on milk yield and composition. It was evaluated in this study by a visual examination of the funnel graphs. The funnel graphs obtained in this meta-analysis were near asymmetry, implying the existence of minimal evidence of publication bias, which could be could be the tendency for negative trials not be published, either because of editorial bias or from authors tending not to be interested in publishing papers with negative results (57). The Nfs for the database are 4 folds above the threshold of 175 and 145 needed to consider the mean effect size robust despite the possibility of publication bias (44). Hence, publication bias was not an issue in this study as a relatively large number of unpublished studies would be needed to alter statistically significant effects.

#### Limitations and strengths of the analysis

This meta-analysis was restricted to studies that investigated the effect of SC products on DMI, milk yield and components in lactating goats and may not apply to other animal species. Few studies were used to assess the impact of SC supplementation on milk ash in lactating goats, and the results should be interpreted with caution. The amount of heterogeneity accounted by diet type, diet composition, age and season of the year the study was conducted was not determined in this meta-analysis because of insufficient data. The influence of SC products on nutrient digestibility in lactating goats was not assessed in the current study due to the small number of identified studies. Despite the observed limitations, the strength of this meta-analysis includes a systematic characterization of uncharacterized studies by aggregating data from studies published in eleven countries by different researchers to increase statistical power, resolve conflicts, identify research gaps and create new insights on the effect of SC on DMI, milk yield and components in lactating goats. This study also defines the guidelines to standardized experimental designs for future experiments.

## Conclusion

The results of this meta-analysis indicate that SC had a moderate positive effect on milk yield and a small effect on DMI and milk components in lactating goats. Subgroup analyses by SC type suggested that dead SC treatment had a moderate negative effect on milk yield and a large reduction effect on DMI. On the other hand, dietary live SC had a large effect on milk yield in lactating goats. This study showed evidence of significant heterogeneity across trials that examined the influence of SC on DMI, milk yield and fat percentage in lactating goats. Meta-regression indicated that prediction variables were significant predictors of the intervention (SC) effect and explained most of the sources of heterogeneity. Furthermore, these findings will help dairy farmers, ruminant nutritionists, and policy-makers make an informed decision about the potential of SC products to improve milk yield and components in lactating goats.

## Data availability statement

The original contributions presented in the study are included in the article/Supplementary material, further inquiries can be directed to the corresponding author/s.

## Author contributions

IO conceptualized and designed the study, collected and analyzed the data, and wrote the initial manuscript. CM visualized the data, reviewed the included articles, and performed the analyses. IO and CM revised the draft. Both authors read and approved the final draft.

## Conflict of interest

The authors declare that the research was conducted in the absence of any commercial or financial relationships that could be construed as a potential conflict of interest.

## Publisher's note

All claims expressed in this article are solely those of the authors and do not necessarily represent those of their affiliated organizations, or those of the publisher, the editors and the reviewers. Any product that may be evaluated in this article, or claim that may be made by its manufacturer, is not guaranteed or endorsed by the publisher.
