# Corrigendum: Meta-analytic effect of *Saccharomyces cerevisiae* on dry matter intake, milk yield and components of lactating goats

**DOI:** 10.3389/fvets.2023.1144334

**Published:** 2023-02-07

**Authors:** Ifeanyi Princewill Ogbuewu, Christian Anayo Mbajiorgu

**Affiliations:** ^1^Department of Animal Science and Technology, Federal University of Technology, Owerri, Imo State, Nigeria; ^2^Department of Agriculture and Animal Health, University of South Africa, Florida, South Africa

**Keywords:** lactating goats, yeast products, supplementation, milk production traits, meta-analysis

In the published article, there was an error in the affiliation of author Christian Anayo Mbajiorgu. The author was incorrectly affiliated to “Department of Animal Science and Technology, Federal University of Technology, Owerri, Imo State, Nigeria”.

The correct affiliation list appears below.

Ifeanyi Princewill Ogbuewu^1,2^^*^, Christian Anayo Mbajiorgu^2^

^1^Department of Animal Science and Technology, Federal University of Technology, Owerri, Imo State, Nigeria

^2^Department of Agriculture and Animal Health, University of South Africa, Florida, South Africa

The authors apologize for this error and state that this does not change the scientific conclusions of the article in any way. The original article has been updated.

