# Supplementary material for: Meta-analytic effect of Saccharomyces cerevisiae on dry matter intake, milk yield and components of lactating goats
Source: Front Vet Sci. 2022 Nov 18;9:1014977. doi: 10.3389/fvets.2022.1014977 (PMC9715603; doi:10.3389/fvets.2022.1014977)
Supplement: Supplementary file 1 [file Data_Sheet_1.docx]

| 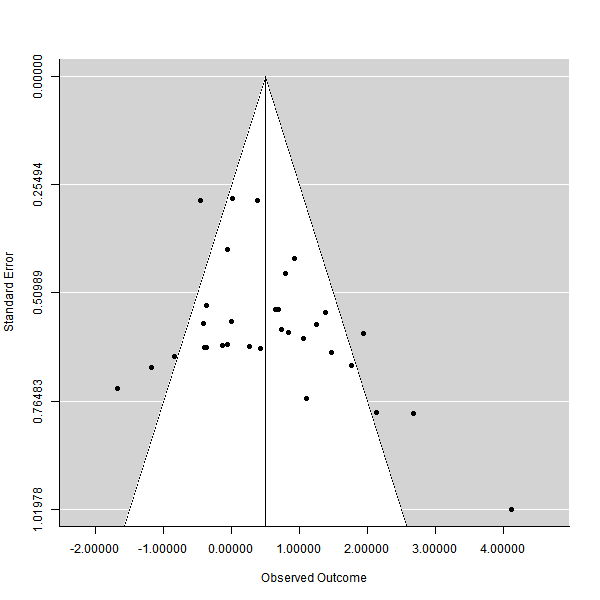 |
| --- |
| Standardized Mean Differences |

**Supplementary Fig. S1.** Funnel plots of the mean effect sizes (SMD) from studies that evaluated milk yield of lactating goats fed diets with and without SC supplementation. The thick vertical solid line represents the summary effect estimates, and the dotted lines are pseudo 95% CIs.

| **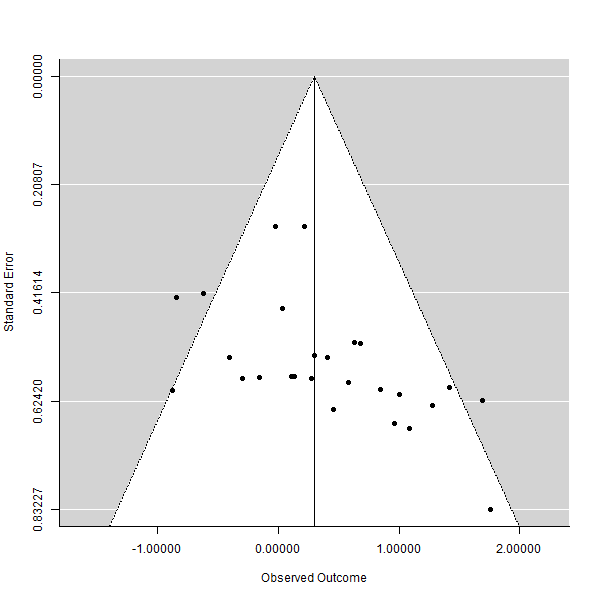** |
| --- |
| Standardized Mean Differences |

**Supplementary Fig. S2.** Funnel plots of the mean effect sizes (SMD) from studies that evaluated milk fat of lactating goats fed diets with and without SC supplementation. The thick vertical solid line represents the summary effect estimates, and the dotted lines are pseudo 95% CIs.
